# On The Protection by The Combination of CeO_2_ Nanoparticles and Sodium Selenite on Human Lymphocytes against Chlorpyrifos-Induced Apoptosis *In Vitro*

**DOI:** 10.22074/cellj.2016.3748

**Published:** 2015-07-11

**Authors:** Sahar Pedram, Azadeh Mohammadirad, Mohammad Amin Rezvanfar, Mona Navaei-Nigjeh, Maryam Baeeri, Mohammad Abdollahi

**Affiliations:** 1 Toxicology and Poisoning Research Center, Department of Toxicology and Pharmacology, Faculty of Pharmacy, Tehran University of Medical Sciences, Tehran, Iran; 2 Faculty of Pharmacy and Pharmaceutical Sciences Research Center, Tehran University of Medical Sciences, Tehran, Iran; 3 Department of Tissue Engineering, School of Advanced Technologies in Medicine, Tehran University of Medical Sciences, Tehran, Iran

**Keywords:** Organophosphorus, Chlorpyrifos, Lymphocytes, Cerium Oxide Nanoparticles, Sodium Selenite

## Abstract

**Objective:**

Chlorpyrifos (CP) as an organophosphorus pesticide is thought to induce oxidative stress in human cells via producing reactive oxygen species (ROS) that leads to the presence of pathologic conditions due to apoptosis along with acetylcholinesterase (AChE) inhibition.This study aimed to evaluate the apoptotic effects of CP and to assess the protective potential of CeO_2_nanoparticle (CNP) and sodium selenite (SSe) by measuring cascades of apoptosis, oxidative stress, inflammation, and AChE inhibition in human isolated lymphocytes.

**Materials and Methods:**

In the present experimental study, we examined the anti-oxidative and AChE activating potential of CNP and SSe in CP-treated human lymphocytes. Therefore, the lymphocytes were isolated and exposed to CP, CP+CNP, CP+SSe, and CP+CNP+SSe after a three-day incubation. Then tumor necrosis factor-alpha (TNF-α) release, myeloperoxidase (MPO) activity, thiobarbituric acid-reactive substances (TBARS) levels as inflammatory/oxidative stress indices along with AChE activity were assessed. In addition, the apoptotic process was measured by flow cytometry.

**Results:**

Results showed a significant reduction in the mortality rate, TNF-α, MPO activity, TBARS, and apoptosis rate in cells treated with CNP, SSe and their combination. Interestingly, both CNP and SSe were able to activate AChE which is inhibited by CP. The results supported the synergistic effect of CNP/SSe combination in the prevention of apoptosis along with oxidative stress and inflammatory cascade.

**Conclusion:**

CP induces apoptosis in isolated human lymphocytes via oxidative stress and inflammatory mediators. CP firstly produces ROS, which leads to membrane phospholipid damage. The beneficial effects of CNP and SSe in reduction of CP-induced apoptosis and restoring AChE inhibition relate to their anti-oxidative potentials.

## Introduction

Pesticides are able to modify immune responses mediated through lymphocytes as found in experimental animals and human subjects poisoned (1). The activation of lymphocytes is a prerequisite for many immunological responses (2). During the last 20 years, several experimental evidences have shown that organophosphorus (OP) compounds can interfere with the immune system and possess immunotoxic effects in the laboratory animal through the lymphocytes and other immunocompetent cells (3), that in case of chronic exposure results in incidence of human diseases (4, 5).

Chlorpyrifos (CP) [0, 0- diethyl 0-(3, 5, 6- tricloro-2- pyridinol) phosphorothionate] is a broad spectrum chlorinated OP insecticide that is now extensively used in the agricultural and residential pest control around the world (6).

As reviewed recently, OPs act through oxidative stress mechanisms (7, 8) and provide major toxicity when the organism is exposed to these compounds for a long time. Unfortunately, excessive use of pesticides in the agriculture by irresponsible persons in the line of higher production has caused the problem of entrance of pesticides into the human food cycle.

Recent studies have indicated that substances with the ability to reduce oxidative stress (9-12) or those having adenosine triphosphate (ATP) donor potentials (13) through mitochondrial mechanisms (14) can reduce toxicity of OPs. Some of antioxidant nanoparticles have been found useful in this respect and are under study of some research groups like the authors of this paper. Therefore, considering the oxidative stress mechanisms of OPs, some nanoparticles such as nanomagnesium (15), nanocerium (16), and nanoselenium (17) have been examined recently. For instance, in the recent years, the efficacy of antioxidant nanoparticles in disease models of colitis (18), diabetes (19), pancreatitis (20, 21), diabetic neuropathy (15), and cardiotoxicity (22, 23), have been proved.

One of these miracle nanoparticles is cerium oxide (CeO_2_, CNP) that is thought to markedly increase the antioxidant power of exposed organs or cells via its major free radical scavenging potential (20).

On the other hand, CNP is able to act like superoxide dismutase (SOD) as a free radical detoxifying system (24). Besides the antioxidant effect, CNP can remain active in the living cells for an extended period of time. Selenium as sodium selenite (SSe) is an essential trace element which possesses a critical role in some protective enzymes against free radicals (25). It also inhibits the adhesive molecules induced by tumor necrosis factor-alpha (TNF-α) and deactivates nuclear factor kappa-light-chain-enhancer of activated B cells (NF-κB) (26). In addition, SSe has been found beneficial in the rats exposed to CP by restoring the oxidative injury (27). SSe in nano or usual form or in combination with other compounds has been found a strong reducer of oxidative stress (10, 25, 27).

Given above evidences, the aim of this study was to evaluate the apoptotic effects of CP and to assess the protective potential of CNP and SSe by measuring cascades of apoptosis, oxidative stress, inflammation, and acetylcholinesterase (AChE) inhibition in human isolated lymphocytes.

## Materials and Methods

### Chemicals

All chemicals were purchased from Sigma- Aldrich Chemie (Germany), whereas CNP was purchased from Navarrean Nanoproducte Technology (Spain), TNF-α ELISA kit was purchased from BenderMed Systems (Austria) and ApoFlowEx® FITC Kit was purchased from Exbio (Czech Republic).

### Lymphocyte isolation and culture

This experimental study was approved by the Institutional Review Board of Tehran University of Medical Sciences with code number of 90-04-151-16052 and all ethical considerations were adhered. Peripheral blood lymphocytes were isolated from heparinized venous blood, which obtained from 10 healthy male volunteers aged between 20-30 years old who were nonsmoker and taking no medications, after obtaining an informed consent from all participants. Blood was mixed with Ficoll-Paque and centrifuged at 400 g for 30 minutes. The lymphocytes were collected from the interface of plasma and Ficoll-Paque, washed three times with phosphate buffered saline (PBS), and then were counted based on the trypan blue exclusion method. After washing and counting, the cells (viability>98%) were cultured (105 cell/ml), in RPMI-1640 consisting of 10% fetal bovine serum (FBS), 2 mM L-glutamine, 100 U/ml penicillin and 100 μg/ml streptomycin sulfate that was followed by addition of 50 μl/ml lipopolysaccharide (LPS) for cell growth stimulation. Cell cultures were grown in 96-well microtiter plates and maintained at 37˚C with 5% CO_2_ humidified atmosphere for 72 hours.

### Chlorpyrifos, CeO_2_ nanoparticle and sodium selenite dose optimization

Before performing the following tests, we determined the cytotoxicity as inhibition concentration (IC50) of CP and the effective doses (ED50) of CNP and SSe in the prevention of CP-induced oxidative stress. It was already shown that CP induces oxidative stress in human erythrocytes [red blood cells (RBCs)] at the average dose of 10 μg/ml (6). In this regard, the cell suspension was incubated with culture medium in combination with 0, 12.5, 25 and 50 μg/ml CP for 72 hours at 37˚C with 5% CO_2_ humidified atmosphere. The suspension was regularly monitored for any sign of contamination or change in the pH.

According to our recent study, for determining the effective doses (ED50) of CNP and SSe in the prevention of CP-induced oxidative stress (16), we used different concentrations of CNP (0, 0.5, 1 and 2 ng/ml) and SSe (0, 0.125, 0.25 and 0.5 ng/ml) based on a pilot study, whichwere incubated at 37˚C with 5% CO_2_ humidified atmosphere in the presence of CP at dose of 12 μg/ml.

### Experimental groups (in vitro)

After determining IC50 of CP and the ED50 of CNP and SSe, all cells were divided into five groups as follows: i. control group, ii. CNP group receiving 1 ng/ml cerium oxide nanoparticles plus 12 μg/ml CP. iii. SSe group receiving 0.36 ng/ml sodium selenite plus 12 μg/ml CP. iv. CNP+SSe group as a combination group receiving 1 ng/ml cerium oxide nanoparticles plus 0.36 ng/ml sodium selenite plus 12 μg/ml CP and v. CP group receiving only 12 μg/ml CP. Then the lymphocytes were incubated at 37˚C with 5% CO_2_ humidified atmosphere. After a 72-hour period, the cell suspensions in all groups were centrifuged at 250 g for 5 minutes. The supernatant solution was removed for the biochemical assays and the precipitated cells were used in methyl thiazolyl tetrazolium (MTT) reduction assay in the next step.

### Lymphocytes viability

The assay is based on the reduction of MTT, a yellow tetrazole, to purple insoluble formazan by mitochondrial respiration in viable cells. MTT assay was performed on human lymphocytes cultured after 72 hours incubation. Centrifugation was done and the precipitated lymphocytes were washed twice with PBS. Then, 30 μl of MTT (5 mg/ml PBS) was added and it was re-incubated for 4 hours at 37˚C with 5% CO_2_ humidified atmosphere. Next, cells were treated with 150 μl of dimethyl sulfoxide (DMSO) and the absorbance was read at 570 nm by enzyme-linked immunosorbent assay (ELISA) reader. For subtracting the MTT background, the absorbance was read at 690 nm in order to reduce artifacts. The viability of the treatment groups was shown as the percentage of controls, assumed to be 100% (16).

### Measurement of thiobarbituric acid-reactive substances as marker of lipid peroxidation

To measure lipid peroxidation, we used TBARS. TBA reactivity of lipid peroxides in the samples produces a measurable pink color that has an absorbance at 532 nm using ultraviolet (UV) spectrophotometer, described in our previous work (28, 29). The activity was shown as μM.

### Measurement of acetylcholinesterase

AChE activity in lymphocytes was assayed according to the modified Ellman method using acetylthiocholine iodide as the substrate and 5-5-bis dithionitrobenzoic acid (DTNB) as coloring agent (30). The activity was expressed as U/ml.

### Measurement of myeloperoxidase activity

To assay MPO activity, we measured it spectrophotometrically as follows: 0.1 ml of supernatant was added to 2.9 ml of 50 mM PBS containing 0.167 mg/ml O-dianisidine hydrochloride and 0.0005% H_2_O_2_. The change in absorbance was recorded by spectrophotometer at 460 nm. MPO activity was defined as the absorbance change per minute at 25˚C in the final reaction (29). The MPO activity was shown in U/ml.

### Measurement of tumor necrosis factor-alpha

A human specific ELISA kit was used to quantify TNF-α in the supernatant of lymphocyte culture. To assess the amount of TNF-α, the absorbance of the sample was measured at 450 nm as the primary wavelength and 620 nm as the reference wavelength by ELISA reader, as described in the manufacturer’s instructions. Data are shown as μg/ml.

### Measurement of apoptosis by flow cytometer

Apoptosis (a programmed cell death) is a well described phenomenon occurring in many cellular systems. Annexin-V staining was assessed by flow cytometer to investigate CP-induced apoptosis (31) pattern. Annexin V binding as an indicator of phosphatidyl serine surface exposure in early apoptotic cells and propidium iodide (PI) staining as necrosis indicator were used. Currently, the most widely used analytical assays are based on monitoring of translocation of phosphatidylserine (PS) from inner phospholipid layer to the cell surface by use of a fluorochrome-labelled Annexin V in combination with appropriate vital dyes. ApoFlowEx® FITC Kit is based on standard setup that employs Annexin V-FITC conjugate and PI. The flow cytometry test can discriminate intact cells (annexin V-/PI), early apoptotic cells (annexin V+/PI), late apoptosis cells (annexin V+/PI+) and necrotic cells (annexin V-/PI+). The precipitated lymphocytes were washed twice with PBS. Then, the cells were suspended in binding buffer at 3×10^5^ cells/100 μl, supplemented with 5 μl of FITC-Annexin-V and 5 μl of PI, and incubated for 15 minutes at room temperature in the dark. Flow cytometric analysis (Apogee, UK) was performed.

### Statistical analysis

Each experiment was carried out at least three times. Data are presented as mean ± standard error of mean (SEM). One-way ANOVA and Tukey’s multiple-comparison tests were carried out by Stats-Direct 3.0.107 to determine the statistical differences, while the level of significance was set at P<0.05.

## Results

### Chlorpyrifos, CeO_2_ nanoparticle and sodium selenite dose optimization

**Fig.1:**
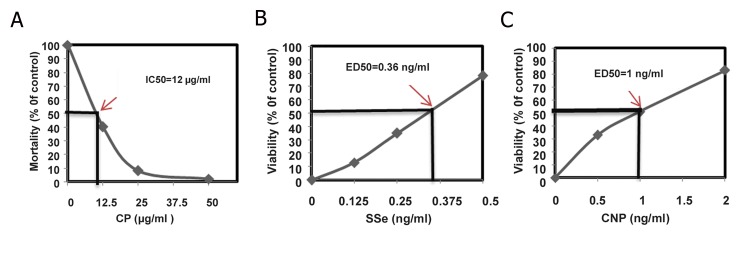
Effects of A. CP, B. SSe and C. CNP on mortality of isolated human lymphocytes. The median inhibitory concentration (IC50) of CP was 12 μg/ml. The cell viability by 50% as the effective dose (ED50) of CNP was 1 ng/ ml and of SSe was 0.36 ng/ml. CP; Chlorpyrifos, SSe; Sodium selenite and CNP; Cerium oxide nanoparticle.

As shown in figure 1A, the MTT reduction assay was used to calculate the median IC50 for CP after 72 hours of exposure (IC50=12 μg/ ml).

After 72 hours, the concentration of CNP that was able to induce the cell viability by 50% was determined by the effective dose of CNP based on MTT reduction assay, as depicted in figure 1B and C (ED50=1 ng/ml). Also, we found the effective dose of SSe that was able to increase the viability to 50% using the MTT reduction assay (ED50=0.36 ng/ml).

### Lymphocytes viability

**Fig.2:**
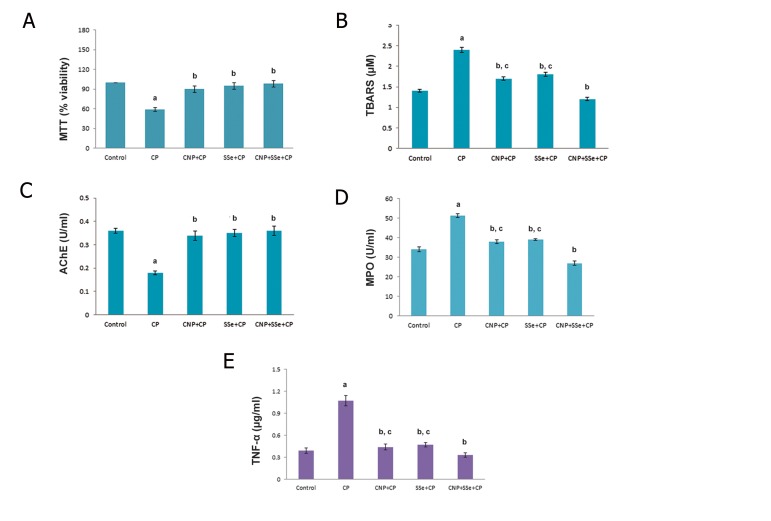
A. Effects of CP, CNP+CP, SSe+CP and CNP+SSe+CP in MTT assay, B. TBARS levels, C. AChE activity, D. MPO activity and E.TNF-α release of isolated human lymphocytes. Data are expressed as mean ± SEM. ^a^; Significant difference between control and other groups, ^b^; Significant difference between CP and other groups and c; Significant difference between CNP+SSe+CP and other groups. CP; Chlorpyrifos , CNP; Cerium oxide nanoparticle, SSe; Sodium selenite, MTT; Methyl thiazolyl tetrazolium, TBARS; Thiobarbituric acidreactive substances, AChE; Acetylcholinesterase, MPO; Myeloperoxidase, TNF-α; Tumor necrosis factor-alpha and SEM; Standard error of mean.

The results of MTT assay on the cultured lymphocyte after 72 hours of different treatments are shown in figure 2A. There is a significant different viability between control and CP (P=0.001) groups. The groups which were pretreated solely with CNP or SSe (P=0.001) remained more viable as compared with the CP group (P=0.001), but didn’t show any different viability compared to the control group. The more improvement in the lymphocyte viability was observed when the cells were pretreated with the combination of CNP+SSe compared to the CP group (P=0.001). Also, the combination group showed no significant decrease in viability and no synergistic effects as compared to CNP (P=0.098) and SSe (P=0.086) groups.

### Thiobarbituric acid-reactive substances levels

As shown in figure 2B, TBARS level was significantly higher in CP group compared to control group (P=0.001). The groups which were pretreated with solely CNP and SSe (P=0.009) showed an apparent reduction in TBARS when compared to CP group (P=0.008), but no differences in comparison to control group. There was a significant decrease in TBARS level of the CNP+SSe group as compared to CP group (P=0.001). Also, the combination group possessed a significant decrease in TBARS showing synergic or additive effects in comparison to CNP (P=0.018) and SSe (P=0.033) groups.

### Acetylcholinesterase activity

As shown in figure 2C, AChE activity was significantly lower in the CP group compared to control group (P=0.001). The groups which were pretreated with solely CNP and SSe showed an apparent increase in AChE activity as compared to CP group (P=0.007 and P=0.001, respectively), but no differences as compared to control group. There was a significant increase in AChE activity in the CNP+SSe group as compared to CP group (P=0.001). The AChE activity improved with both CNP and SSe, especially their combination, but no synergistic or additive effects were observed.

### Myeloperoxidase activity

As depicted in figure 2D, MPO activity increased in the CP group as compared to the control group (P=0.001). The CNP- and SSe-pretreated cells showed a significant decrease in MPO activity in comparison to CP group (P=0.004 and P=0.007, respectively). The CNP+SSe group reduced the MPO activity as compared with the CP group (P=0.001). This combination showed synergistic or additive effects in comparison to CNP (P=0.002) and SSe (P=0.004) alone in lowering the MPO activity.

### Tumor necrosis factor-alpha release

As seen in figure 2E, TNF-α production significantly elevated in the CP group when compared to the control group (P=0.001). A significant decrease in TNF-α levels was seen in the CNP and SSe treatment groups as compared to CP group (P=0.001 and P=0.001, respectively). The combination group showed more reduction in TNF-α protein production, compared to CP group (P=0.001). Also, CNP+SSe group showed a significant decrease in TNF-α levels as well as a synergistic or additive effect in comparison to CNP (P=0.024) and SSe (P=0.039) groups.

### Annexin V staining and flow cytometry analysis

**Fig.3:**
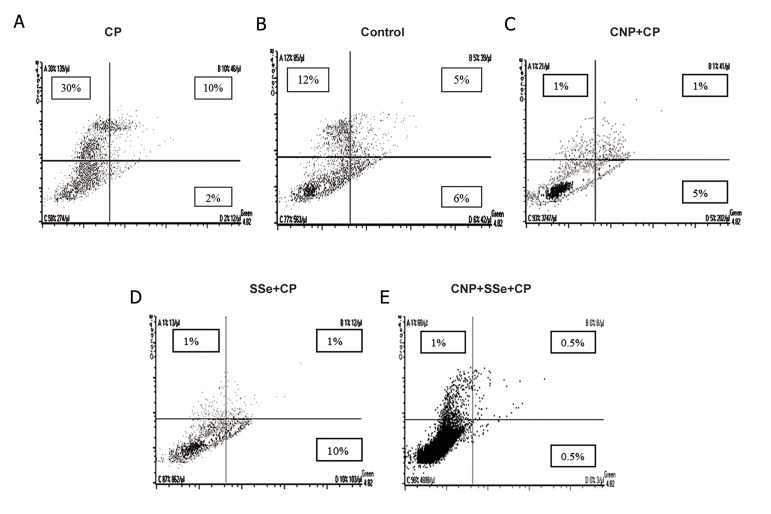
Effects of CP, CNP+CP, SSe+CP and CNP+SSe+CP in apoptosis of isolated human lymphocytes. Data are expressed as mean ± SEM. CP; Chlorpyrifos, SSe; Sodium selenite, CNP; Cerium oxide nanoparticle and SEM; Standard error of mean.

As shown in figure 3, all treated groups, pluscontrol group appeared to contain a significantly lower percentage of apoptotic cells in comparison to the CP group.

We found that in CP-treated cells, 2% were annexin V+/PI-, 10% were annexin V+/PI+ and 30% were annexin V-/PI+ (Fig.3A).

Among the control cells, only 6% were annexin V+/PI-, 5% were annexin V+/PI+ and 12% were annexin V-/PI+ (Fig.3B).

In comparison to CP-treated cells, annexin V+/PI- cells increased to 5% and 10.3% after being treated with CNP and SSe, respectively (Fig.3C, D), although it decreased to 0.5% after being treated with their combination (Fig.3E).

Also, as compared with CP-treated cells, the annexin V+/PI+ cells decreased to 1, 1 and 0.5% after being treated with CNP, SSe and their combination, respectively (Fig.3C-E).

In addition, in comparison with CP-treated cells, annexin V-/PI+ cells decreased to 1, 1 and 1% after being treated with CNP, SSe and their combination, respectively (Fig.3C-E).

Interestingly, this combination showed synergistic or additive effects in comparison to CNP and SSe alone in reduction of early apoptosis or annexin V+/PI- cells. Also, the results of late apoptosis or annexin V+/PI+ cells confirmed synergistic or additive effects of combination as compared to CNP and SSe alone.

**Table 1:** Effects of CP, CNP+CP, SSe+CP and CNP+SSe+CP on MTT assay, TBARS levels, AChE activity, MPO activity, and TNF-α release of isolated human lymphocytes


	MTT(%viability)	P value	TBARS(µM)	P value	AChE (U/ml)	P value	MPO(U/ml)	P value	TNF-alpha(pg/ml)	P value

Control	100 ± 1	-	1.4± 0.034	-	0.36± 0.034	-	34± 1.2		0.39± 0.04	
CP	59+2.8^a^	0.001	2.4± 0.06^a^	0.001	0.18± 0.06^a^	0.001	51.2 ± 0.99^a^	0.001	1.07± 0.07^a^	0.001
CNP+CP	90 ± 4.8^b^	0.001	1.7± 0.04^b^^,^^c^	0.009, 0.018	0.35± 0.043^b^	0.007	38± 0.97^b^^,^^c^	0.004, 0.002	0.44± 0.04^b^^,^^c^	0.001, 0.024
SSe+CP	95 ± 4.8^b^	0.001	1.8± 0.05^b^^,^^c^	0.008, 0.033	0.34± 0.038^b^	0.001	39± 0.42^b^^,^^c^	0.007, 0.004	0.47± 0.03^b^^,^^c^	0.001, 0.039
CNP+SSe+CP	98 ± 5.2^b^	0.001	1.2± 0.04^b^^,^^c^	0.001	0.36± 0.048^b^	0.001	27± 1.03^b^	0.001	0.33± 0.03^b^^,^^c^	0.001


Data are expressed as mean±SEM. ^a^; Significant difference between control and other groups. ^b^; Significant difference between CP and other groups and ^c^; Significantly different from CNP+SSe+CP and other groups. CP; Chlorpyrifos, CNP; Cerium oxide nanoparticle, SSe; Sodium selenite, MTT; Methyl thiazolyl tetrazolium, TBARS; Thiobarbituric acidreactive substances, AChE; Acetylcholineesterase, MPO; Myeloperoxidase, TNF-α; Tumor necrosis factor-alpha and SEM; Standard error of mean.

## Discussion

The goal of the present study was to investigate one of the widely used pesticides CP for its potential to induce apoptosis and oxidative stress in the isolated human lymphocytes and also to evaluate the protective effects of CNP and SSe, especially their combination (Table 1). The results of this study showed that incubation with CP significantly increased levels of oxidative stress and key inflammatory biomarkers such as TBARS, MPO, and TNF-α, whereas their activities were prevented when CNP, SSe or their combination were used. Although, the AChE activity improved with both CNP and SSe, especially their combination, no synergistic or additive effects were observed.

Moreover, lymphocytes treated with CNP, SSe, and their combination, resulted in a significant decrease in the percent of mortality and apoptotic cells, inflammatory as well as oxidative markers.

It has been reported that Annexin-V staining is able to detect apoptosis in the early stage based on alterations of the cell membrane (31). In this regard, our findings of Annexin-V staining assay indicated that CP-induced cell death consisted of apoptosis in human lymphocytes. In support of this result, it has been reported that CP induces apoptosis in human natural killer (NK) cells (32), human monocyte cell line U937 (33), and a murine EL4 T-lymphocytic leukemia cell line (34).

It has been well established that OP causes mitochondrial damage and dysfunction due to increased generation of ROS, induction of proteolytic enzymes, and apoptotic death (35). In addition, the phospholipid component of cell membranes is suggested as a site of toxic action of OP compound (14). Exposure of phospholipids on the external surface of the cell membrane has been reported for activated apoptosis process. On the other hand, apoptosis is a cell death process characterized by specific features occurring at different stages. At a stage of early apoptosis (annexin V+/PI), translocation of phospholipid phosphatidylserine and reduction of apoptosis occurs, whereas on stage of late apoptosis cells (annexin V+/PI+), the cells are already dead and phospholipid translocation has already occurred; therefore, use of CNP and SSe combination is better than that of CNP or SSe alone. So, the results are in favor of synergism or additive effects of combination.

Inhibition of AChE as a main mechanism of toxicity of CP is related to the cell membrane of lymphocytes and monocytes (36) that may lead to structural or functional alterations in immunocyte populations. Treatment of CNP, SSe and their combination alleviated AChE inhibitory activity in the CP-induced human lymphocytes, demonstrating that alteration of AChE activity might be the down stream effect of oxidative stress. Although, this is the first study targeting the effects of CNP in increasing AChE activity, there are several evidences about potential of SSe in augmentation of AChE activity (37). This element is capable of improving the toxicity of cadmium, mercury and lead which induced AChE inhibition in fish brain (38). On the other hand, supplementation with SSe (0.05 mg/kg/day) produced a beneficial effect on the buffalo calves intoxicated with CP (39).

In our study, CP caused lipid damage as indicated by the rise of TBARS, the marker of lipid peroxidation, resulting from the direct interaction of ROS and unsaturated fatty acids (40). This increase was associated with the protection provided by CNP, SSe and their combination. In support of the present findings, available reports have exhibited that CP increases TBARS in the erythrocytes (*in vivo* and *in vitro*) as well as in the brain, lung, testes, kidney and the liver (41-43). MPO, a heme protein, performs as an oxidant enzyme in the process of inflammation and generates reactive intermediates that progress lipid peroxidation in vitro (44). Interestingly, in this study, the results confirmed an increase in the TBARS which was associated with an enhancement of MPO activity in the lymphocytes treated with CP. In addition, this result is supported by our previous reports of *in vitro* effects of CNP, SSe and their combination, as anti-oxidative agents on isolated rat islets (16). Moreover, CNP has been reported to diminish oxidative signaling and cell mortality induced by cigarette smoke, diesel exhaust, and hydrogen peroxide (45-47). In addition, SSe has been found beneficial in the rats exposed to CP by restoring the oxidative injury (27).

Cytokines, regulators of immuneresponses play an important role in activation, proliferation and differentiation of lymphocytes in response to pesticide exposure (48). Release of TNF-α from human blood mononuclear cells, following an immunologic response, is an index of the inflammatory processes which may result in the peroxidation of cell proteins, lipids and cell apoptosis (49). Our data supported previous studies showing that TNF-α levels increase in animals exposed to CP (50), while interestingly showed the protective effects of CNP, SSe and their combination in reduction of TNF-α in CP-treated lymphocytes. The anti-inflammatory effects of CNP in macrophages showed its effect by reduction of inducible nitric oxide expression (46). Also, inflammatory factors were reduced by CNP in a murine cardiomyopathy model (51). SSe, as an essential trace element, possesses a critical role in some protective enzymes against free radicals (25), inhibits the adhesive molecules induced by TNF-α, and deactivates NF-κB (52).

The results of MTT assay suggest that CP disrupts mitochondrial function, showing involvement of the mitochondrial pathway (33). In addition, our result of the protective effect of CNP, SSe and their combination, is supported by our previous reports of *in vitro* effects of these elements, as antioxidant agents on isolated rat islets (19). It has been reported that CP induces apoptosis in rat neurons via a balanced mechanism regulated by p38 mitogen-activated protein (MAP) kinases, extracellular signal-regulated protein kinase (ERK), and c-Jun NH_2_-terminal protein kinase (JNK) (53). Further studies are necessary to explore the detailed effect of CP on the mitochondrial pathway.

## Conclusion

Our results demonstrated that CP induces apoptosis in isolated human lymphocytes via oxidative stress and inflammatory mediators. This kind of apoptosis in lymphocytes would without doubt affect its function and can be named immunotoxicity, although it is not new for OP compounds. It seems that CP firstly produces ROS, which leads to membrane phospholipid damage. The beneficial effects of CNP and SSe in reduction of CP-induced apoptosis and restoring AChE inhibition are related to their antioxidant potentials. Therefore, application of the CNP and SSe combination is reasonable in protection of toxic effects of CP. Of course, this remains to be further examined *in vivo* and in the clinic.

## References

[1] Seth V, Banerjee BD, Bhattacharya A, Pasha ST, Chakravorty AK (2001). Pesticide induced alterations in acetylcholine esterase and gamma glutamyl transpeptidase activities and glutathione level in lymphocytes of human poisoning cases. Clin Biochem.

[2] Galloway T, Handy R (2003). Immunotoxicity of organophosphorous pesticides. Ecotoxicology.

[3] Corsini E, Liesivuori J, Vergieva T, VanLoveren H, Colosio C (2008). Effects of pesticide exposure on the human immune system. Hum Exp Toxicol.

[4] Mostafalou S, Abdollahi M (2013). Pesticides and human chronic diseases: evidences, mechanisms, and perspectives. Toxicol Appl Pharmacol.

[5] Abdollahi M, Karami-Mohajeri S (2010). A comprehensive review on experimental and clinical findings in intermediate syndrome caused by organophosphate poisoning. Toxicol Appl Pharmacol.

[6] Shankarjit S, Aruna B (2013). Biotherapeutic potential of Ziziphus mauritiana (Lamk.) extract against chlorpyrifos induced oxidative stress (an in-vitro study). Biochem Mol Biol.

[7] Abdollahi M, Ranjbar A, Shadnia S, Nikfar S, Rezaiee A (2004). Pesticides and oxidative stress: a review. Med Sci Monit.

[8] Soltaninejad K, Abdollahi M (2009). Current opinion on the science of organophosphate pesticides and toxic stress: a systematic review. Med Sci Monit.

[9] Hosseini A, Baeeri M, Rahimifard M, Navaei-Nigjeh M, Mohammadirad A, Pourkhalili N (2013). Antiapoptotic effects of cerium oxide and yttrium oxide nanoparticles in isolated rat pancreatic islets. Hum Exp Toxicol.

[10] Baeeri M, Shariatpanahi M, Baghaei A, Ghasemi-Niri SF, Mohammadi H, Mohammadirad A (2013). On the benefit of magnetic magnesium nanocarrier in cardiovascular toxicity of aluminum phosphide. Toxicol Ind Health.

[11] Shadnia S, Ashrafivand S, Mostafalou S, Abdollahi M (2011). N-acetylcysteine a novel treatment for acute human organophosphate poisoning. Int J Pharmacol.

[12] Amirkabirian N, Teimouri F, Esmaily H, Mohammadirad A, Aliahmadi A, Abdollahi M (2007). Protection by pentoxifylline of diazinon-induced toxic stress in rat liver and muscle. Toxicol Mech Methods.

[13] Salari P, Abdollahi M (2009). Current opinion in the pharmaceutical management of irritable and inflammatory bowel diseases: role of ATP. Recent Pat Endocr Metab Immune Drug Discov.

[14] Karami-Mohajeri S, Abdollahi M (2013). Mitochondrial dysfunction and organophosphorus compounds. Toxicol Appl Pharmacol.

[15] Hosseini A, Abdollahi M, Hassanzadeh G, Rezayat M, Hassani S, Pourkhalili N (2011). Protective effect of magnesium-25 carrying porphyrin-fullerene nanoparticles on degeneration of dorsal root ganglion neurons and motor function in experimental diabetic neuropathy. Basic Clin Pharmacol Toxicol.

[16] Pourkhalili N, Hosseini A, Nili-Ahmadabadi A, Rahimifard M, Navaei-Nigjeh M, Hassani S (2012). Improvement of isolated rat pancreatic islets function by combination of cerium oxide nanoparticles/sodium selenite through reduction of oxidative stress. Toxicol Mech Methods.

[17] Rezvanfar MA, Rezvanfar MA, Shahverdi AR, Ahmadi A, Baeeri M, Mohammadirad A (2013). Protection of cisplatininduced spermatotoxicity, DNA damage and chromatin abnormality by selenium nano-particles. Toxicol Appl Pharmacol.

[18] Saadatzadeh A, Atyabi F, Fazeli MR, Dinarvand R, Jamalifar H, Abdolghaffari AH (2012). Biochemical and pathological evidences on the benefit of a new biodegradable nanoparticles of probiotic extract in murine colitis. Fundam Clin Pharmacol.

[19] Navaei-Nigjeh M, Rahimifard M, Pourkhalili N, Nili-Ahmadabadi A, Pakzad M, Baeeri M (2012). Multi-organ protective effects of cerium oxide nanoparticle/selenium in diabetic rats: evidence for more efficiency of nanocerium in comparison to metal form of cerium. Asian J Anim Vet Adv.

[20] Hosseini A, Abdollahi M (2012). Through a mechanism-based approach, nanoparticles of cerium and yttrium may improve the outcome of pancreatic islet isolation. J Med Hypotheses Ideas.

[21] Pourkhalili N, Hosseini A, Nili-Ahmadabadi A, Hassani S, Pakzad M, Baeeri M (2011). Biochemical and cellular evidence of the benefit of a combination of cerium oxide nanoparticles and selenium to diabetic rats. World J Diabetes.

[22] Mohammadi H, Karimi G, Rezayat SM, Dehpour AR, Shafiee H, Nikfar S (2011). Benefit of nanocarrier of magnetic magnesium in rat malathion-induced toxicity and cardiac failure using non-invasive monitoring of electrocardiogram and blood pressure. Toxicol Ind Health.

[23] Shafiee H, Mohammadi H, Rezayat SM, Hosseini A, Baeeri M, Hassani S (2010). Prevention of malathion-induced depletion of cardiac cells mitochondrial energy and free radical damage by a magnetic magnesium carrying nanoparticle. Toxicol Mech Methods.

[24] Hosseini A, Sharifi AM, Abdollahi M, Najafi R, Baeeri M, Rayegan S (2015). Cerium and yttrium oxide nanoparticles against lead-induced oxidative stress and apoptosis in rat hippocampus. Biol Trace Elem Res.

[25] Kohrle J, Jakob F, Contempre B, Dumont JE (2005). Selenium, the thyroid, and the endocrine system. Endocr Rev.

[26] Esmaily H, Vaziri-Bami A, Miroliaee AE, Baeeri M, Abdollahi M (2011). The correlation between NF-κB inhibition and disease activity by coadministration of silibinin and ursodeoxycholic acid in experimental colitis. Fundam Clin Pharmacol.

[27] Heikal TM, El-Sherbiny M, Hassan SA, Arafa A, Ghanem HZ (2010). Antioxidant effects of selenium on hepatotoxicity induced by chlorpyrifos in male rats. Int J Pharm Pharm Sci.

[28] Astaneie F, Afshari M, Mojtahedi A, Mostafalou S, Zamani MJ, Larijani B (2005). Total antioxidant capacity and levels of epidermal growth factor and nitric oxide in blood and saliva of insulin-dependentdiabetic patients. Arch Med Res.

[29] Ghazanfari G, Minaie B, Yasa N, Nakhai LA, Mohammadirad A, Nikfar S (2006). Biochemical and histopathological evidences for beneficial effects of Satureja khuzestanicajamzad essential oil on the mouse model of inflammatory bowel diseases. Toxicol Mech Methods.

[30] Ellman GL, Courtney KD, Andres V Jr, Featherstone RM (1961). A new and rapid colorimetric determination of acetylcholinesterase activity. Biochem Pharmacol.

[31] Dong F, Zhang X, Li SY, Zhang Z, Ren Q, Culver B (2005). Possible involvement of NADPH oxidase and JNK in homocysteine-induced oxidative stress and apoptosis in human umbilical vein endothelial cells. Cardiovasc Toxicol.

[32] Li Q, Kobayashi M, Kawada T (2007). Organophosphorus pesticides induce apoptosis in human NK cells. Toxicology.

[33] Nakadai A, Li Q, Kawada T (2006). Chlorpyrifos induces apoptosis in human monocyte cell line U937. Toxicology.

[34] Saleh AM, Vijayasarathy C, Masoud L, Kumar L, Shahin A, Kambal A (2003). Paraoxon induces apoptosis in EL4 cells via activation of mitochondrial pathways. Toxicol Appl Pharmacol.

[35] Akhgari M, Abdollahi M, Kebryaeezadeh A, Hosseini R, Sabzevari O (2003). Biochemical evidence for free radical-induced lipid peroxidation as a mechanism for subchronic toxicity of malathion in blood and liver of rats. Hum Exp- Toxicol.

[36] Jokanovic M, Stepanovic RM, Maksimovic M, Kosanovic M, Stojiljkovic MP (1998). Modification of the rate of aging of diisopropylfluorophosphate-inhibited neuropathy target esterase of hen brain. Toxicol Lett.

[37] Nehru B, Dua R (1997). The effect of dietary selenium on lead neurotoxicity. J Env Pathol Toxicol Oncol.

[38] Chen MH, Shih CC, Chou CL, Chou LS (2002). Mercury, organicmercury and selenium in small cetaceans in Taiwanese waters. Mar Pollut Bull.

[39] Kaur R, Sandhu HS (2008). In vivo changes in antioxidant system and protective role of selenium in chlorpyrifosinduced subchronic toxicity in bubalus bubalis. Environ Toxicol Pharmacol.

[40] Yu LH, Liu GT, Sun YM, Zhang HY (2004). Antioxidative effect of schisanhenol on human low density lipoprotein and its quantum chemical calculation. Acta Pharmacol Sin.

[41] Karaoz E, Gultekin F, Akdogan M, Oncu M, Gokcimen A (2002). Protective role of melatonin and a combination of vitamin C and vitamin E on lung toxicity induced by chlorpyrifosethyl in rats. Exp Toxicol Pathol.

[42] Oncu M, Gultekin F, Karaoz E, Altuntas I, Delibas N (2002). Nephrotoxicity in rats induced by chlorpryfos-ethyl and ameliorating effects of antioxidants. Hum Exp Toxicol.

[43] Gultekin F, Delibas N, Yasar S, Kilinc I (2001). In vivo changes in antioxidant systems and protective role of melatonin and a combination of vitamin C and vitamin E on oxidative damage in erythrocytes induced by chlorpyrifos-ethyl in rats. Arch Toxicol.

[44] Zhang R, Brennan ML, Shen Z, MacPherson JC, Schmitt D, Molenda CE (2002). Myeloperoxidase functions as a major enzymatic catalyst for initiation of lipid peroxidation at sites of inflammation. J Biol Chem.

[45] Celardo I, Pedersen JZ, Traversa E, Ghibelli L (2011). Pharmacological potential of cerium oxide nanoparticles. Nanoscale.

[46] Hirst SM, Karakoti AS, Tyler RD, Sriranganathan N, Seal S, Reilly CM (2009). Anti-inflammatory properties of cerium oxide nanoparticles. Small.

[47] Xia T, Kovochich M, Liong M, Madler L, Gilbert B, Shi H (2008). Comparison of the mechanism of toxicity of zinc oxide and cerium oxide nanoparticles based on dissolution and oxidative stress properties. ACS Nano.

[48] Corsini E, Codeca I, Mangiaratti S, Birindelli S, Minoia C, Turci R (2007). Immunomodulatory effects of the herbicide propanil on cytokine production in humans: in vivo and in vitro exposure. Toxicol Appl Pharmacol.

[49] Djavaheri-Mergny M, Javelaud D, Wietzerbin J, Besancon F (2004). NF-kappaB activation prevents apoptotic oxidative stress via an increase of both thioredoxin and MnSOD levels in TNFalpha-treated Ewing sarcoma cells. FEBS Lett.

[50] Rowsey PJ, Gordon CJ (1999). Tumor necrosis factor is involved in chlorpyrifos--induced changes in core temperature in the female rat. Toxicol Lett.

[51] Niu J, Wang K, Kolattukudy PE (2011). Cerium oxide nanoparticles inhibit oxidative stress and nuclear factor-κB activation in H9c2 cardiomyocytes exposed to cigarette smoke extract. J Pharmacol Exp Ther.

[52] Esmaily H, Hosseini-Tabatabaei A, Rahimian R, Khorasani R, Baeeri M, Barazesh-Morgani A (2009). On the benefits of silymarin in murine colitis by improving balance of destructive cytokines and reduction of toxic stress in the bowel cells. Cent Eur J Biol.

[53] Caughlan A, Newhouse K, Namgung U, Xia Z (2004). Chlorpyrifos induces apoptosis in rat cortical neurons that is regulated by a balance between p38 and ERK/JNK MAP kinases. Toxicol Sci.

